# Trends and Geographic Patterns in Drug and Synthetic Opioid Overdose Deaths — United States, 2013–2019

**DOI:** 10.15585/mmwr.mm7006a4

**Published:** 2021-02-12

**Authors:** Christine L. Mattson, Lauren J. Tanz, Kelly Quinn, Mbabazi Kariisa, Priyam Patel, Nicole L. Davis

**Affiliations:** ^1^Division of Overdose Prevention, National Center for Injury Prevention and Control, CDC; ^2^Oak Ridge Institute for Science and Education, Oak Ridge, Tennessee.

Deaths involving synthetic opioids other than methadone (synthetic opioids), which largely consist of illicitly manufactured fentanyl; psychostimulants with abuse potential (e.g., methamphetamine); and cocaine have increased in recent years, particularly since 2013 (*1*,*2*). In 2019, a total of 70,630 drug overdose deaths occurred, corresponding to an age-adjusted rate of 21.6 per 100,000 population and a 4.3% increase from the 2018 rate (20.7) (*3*). CDC analyzed trends in age-adjusted overdose death rates involving synthetic opioids, psychostimulants, cocaine, heroin, and prescription opioids during 2013–2019, as well as geographic patterns in synthetic opioid- and psychostimulant-involved deaths during 2018–2019. From 2013 to 2019, the synthetic opioid-involved death rate increased 1,040%, from 1.0 to 11.4 per 100,000 age-adjusted (3,105 to 36,359). The psychostimulant-involved death rate increased 317%, from 1.2 (3,627) in 2013 to 5.0 (16,167) in 2019. In the presence of synthetic opioid coinvolvement, death rates for prescription opioids, heroin, psychostimulants, and cocaine increased. In the absence of synthetic opioid coinvolvement, death rates increased only for psychostimulants and cocaine. From 2018 to 2019, the largest relative increase in the synthetic opioid-involved death rate occurred in the West (67.9%), and the largest relative increase in the psychostimulant-involved death rate occurred in the Northeast (43.8%); these increases represent important changes in the geographic distribution of drug overdose deaths. Evidence-based prevention and response strategies including substance use disorder treatment and overdose prevention and response efforts focused on polysubstance use must be adapted to address the evolving drug overdose epidemic.

Drug overdose deaths were identified in the National Vital Statistics System multiple cause-of-death mortality files* by using *International Classification of Diseases, Tenth Revision (ICD-10)* underlying cause-of-death codes X40–44 (unintentional), X60–64 (suicide), X85 (homicide), or Y10–14 (undetermined intent). Drug categories were defined using the following ICD-10 multiple cause-of-death codes: synthetic opioids other than methadone (T40.4), psychostimulants with abuse potential (T43.6), cocaine (T40.5), prescription opioids (T40.2 or T40.3), and heroin (T40.1). Deaths involving more than one type of drug were included in the rates for each applicable drug category; categories are not mutually exclusive.^†^

Annual age-adjusted death rates^§^ were examined during 2013–2019 and stratified by drug category and synthetic opioid coinvolvement. The percentage of 2019 drug overdose deaths and change in 2018–2019 age-adjusted death rates involving synthetic opioids and psychostimulants were examined by U.S Census region^¶^ and state. States with inadequate drug specificity, too few deaths to calculate stable estimates, or too few deaths to meet confidentiality requirements were excluded from state-level analyses.**^,††^ Analyses of rate changes used z-tests when deaths were ≥100 and nonoverlapping confidence intervals based on a gamma distribution when deaths were <100.^§§^ Changes presented in text represent statistically significant (p<0.05) findings unless otherwise specified. Statistical analyses were conducted in SAS (version 9.4; SAS Institute) and maps were created using QGIS (version 3.4.11-Madeira; QGIS Association).

In 2019, a total of 70,630 drug overdose deaths occurred in the United States, corresponding to an age-adjusted rate of 21.6 per 100,000 population and a 56.5% increase above the 2013 rate of 13.8. From 2013 to 2019, the synthetic opioid-involved death rate increased 1,040%, from 1.0 to 11.4 per 100,000 age-adjusted (3,105 to 36,359) (Figure 1). The psychostimulant-involved death rate increased 317%, from 1.2 (3,627) in 2013 to 5.0 (16,167) in 2019. Smaller but meaningful increases were observed during this period for cocaine (206%; 1.6 to 4.9) and heroin (63%; 2.7 to 4.4). The prescription opioid-involved death rate decreased 4.5% from 4.4 in 2013 to 4.2 in 2019.

**FIGURE 1:**
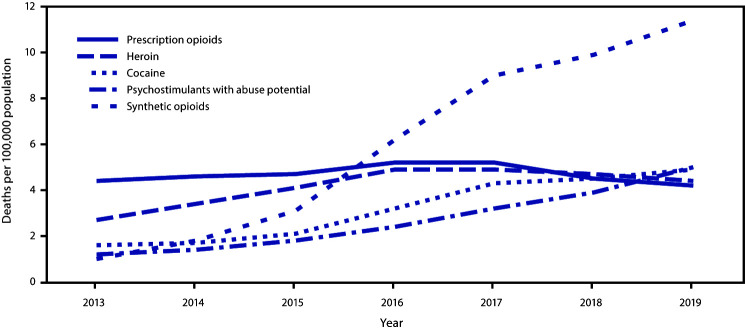
Age-adjusted rates* of drug overdose deaths^†^ involving prescription opioids,^§^ heroin,^¶^ cocaine,** psychostimulants with abuse potential,^††^ and synthetic opioids other than methadone^§§^^,^^¶¶^ — United States, 2013–2019 **Source:** National Vital Statistics System, Mortality File. https://wonder.cdc.gov/ * Rate per 100,000 population age-adjusted to the 2000 U.S. standard population using the vintage year population of the data year. ^†^ Deaths were classified using the *International Classification of Diseases, Tenth Revision*. Drug overdoses are identified using underlying cause-of-death codes X40–X44 (unintentional), X60–X64 (suicide), X85 (homicide), and Y10–Y14 (undetermined). ^§^ Drug overdose deaths, as defined, that involve natural and semisynthetic opioids (T40.2) or methadone (T40.3). ^¶^ Drug overdose deaths, as defined, that involve heroin (T40.1). ** Drug overdose deaths, as defined, that involve cocaine (T40.5). ^††^ Drug overdose deaths, as defined, that involve psychostimulants with abuse potential (T43.6). ^§§^ Drug overdose deaths, as defined, that involve synthetic opioids other than methadone (T40.4). ^¶¶^ Because deaths might involve more than one drug, some deaths are included in more than one category. In 2019, 6.3% of drug overdose deaths did not include information on the specific type of drug(s) involved.

In the presence of synthetic opioid coinvolvement, age-adjusted death rates for all drug categories increased from 2013 to 2019: psychostimulants (0.1 to 1.8), cocaine (0.1 to 3.2), heroin (0.1 to 2.7) and prescription opioids (0.3 to 1.8) (Figure 2). In the absence of synthetic opioid coinvolvement, the age-adjusted death rate increased from 2013 to 2019 for psychostimulants (1.1 to 3.2) and cocaine (1.5 to 1.7); however, rates decreased for prescription opioid- (4.1 to 2.4) and heroin-involved deaths (2.6 to 1.6).

**FIGURE 2:**
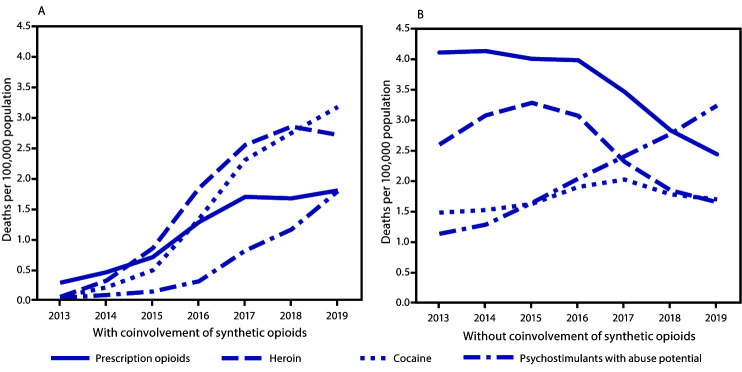
Age-adjusted rates* of drug overdose deaths^†^ involving prescription opioids,^§^ heroin,^¶^ cocaine,** and psychostimulants with abuse potential,^††^ with (A) and without (B) synthetic opioids other than methadone^§§^^,^^¶¶^ — United States, 2013–2019 **Source:** National Vital Statistics System, Mortality File. https://wonder.cdc.gov/ * Rate per 100,000 population age-adjusted to the 2000 U.S. standard population using the vintage year population of the data year. ^†^ Deaths were classified using the *International Classification of Diseases, Tenth Revision*. Drug overdoses are identified using underlying cause-of-death codes X40–X44 (unintentional), X60–X64 (suicide), X85 (homicide), and Y10–Y14 (undetermined). ^§^ Drug overdose deaths, as defined, that involve natural and semisynthetic opioids (T40.2) or methadone (T40.3). ^¶^ Drug overdose deaths, as defined, that involve heroin (T40.1). ** Drug overdose deaths, as defined, that involve cocaine (T40.5). ^††^ Drug overdose deaths, as defined, that involve psychostimulants with abuse potential (T43.6). ^§§^ Drug overdose deaths, as defined, that involve synthetic opioids other than methadone (T40.4). ^¶¶^ Because deaths might involve more than one drug, some deaths are included in more than one category. In 2019, 6.3% of drug overdose deaths did not include information on the specific type of drug(s) involved.

In 2019, a total of 49,860 (70.6%) drug overdose deaths involved opioids, 36,359 (51.5%) involved synthetic opioids, and 16,167 (22.9%) involved psychostimulants. The percentage of drug overdose deaths that involved synthetic opioids was highest in the Northeast (71.0%) and lowest in the West (26.4%). In nine states, ≥70% of overdose deaths involved synthetic opioids (Figure 3); the percentage was highest in New Hampshire (84.3%).

**FIGURE 3:**
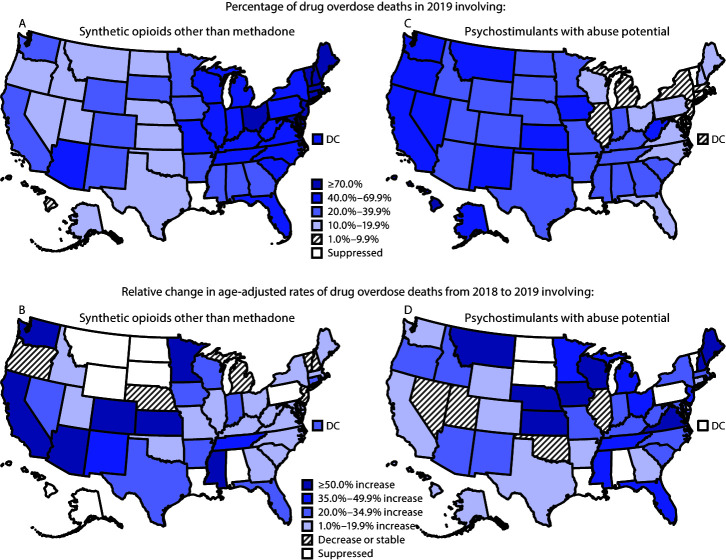
Percentage* and relative change in age-adjusted rates^†^^,^^§^^,^^¶^^,^** of drug overdose deaths^††^ involving synthetic opioids other than methadone (A, B)^§§^ and psychostimulants with abuse potential (C, D)^¶¶^^,^*** — United States, 2018–2019 **Source:** National Vital Statistics System, Mortality File. https://wonder.cdc.gov/ **Abbreviation:** DC = District of Columbia. *State-level analyses of the percentage of drug overdose deaths involving synthetic opioids excluded one state and involving psychostimulants excluded two states that did not meet the following criteria: >80% of drug overdose death certificates named at least one specific drug in 2019 and ≥10 deaths occurred in 2019 in the specific drug category. ^†^ Rate per 100,000 population age-adjusted to the 2000 U.S. standard population using the vintage year population of the data year. ^§^ Z-tests were used if the number of deaths was ≥100 in both 2018 and 2019, and p<0.05 was statistically significant. Nonoverlapping confidence intervals (CIs) based on the gamma method were used if the number of deaths was <100 in 2018 or 2019. The method of comparing CIs is a conservative method for statistical significance; caution should be observed when interpreting a nonsignificant difference when the lower and upper limits being compared overlap only slightly. https://www.cdc.gov/nchs/data/NVSR/NVSR61/NVSR61_04.pdf ^¶^ States with a statistically significant change in age-adjusted rate of drug overdose deaths involving synthetic opioids other than methadone during 2018–2019 were Arizona, California, Colorado, Connecticut, District of Columbia, Florida, Georgia, Illinois, Indiana, Kentucky, Minnesota, Mississippi, New Mexico, New York, Ohio, Tennessee, Texas, Virginia, Washington, and Wisconsin. States with a statistically significant change in age-adjusted rate of drug overdose deaths involving psychostimulants with abuse potential during 2018–2019 were Arizona, California, Florida, Indiana, Iowa, Kansas, Kentucky, Maine, Michigan, Minnesota, Missouri, New Jersey, New Mexico, New York, North Carolina, Ohio, Oregon, South Carolina, Tennessee, Texas, Virginia, Washington, West Virginia, and Wisconsin. ** State-level analyses comparing death rates from 2018 to 2019 excluded nine states that did not meet the following criteria: >80% of drug overdose death certificates named at least one specific drug in 2018 and 2019 and ≥20 deaths occurred during 2018 and 2019 in the drug category examined. ^††^ Deaths were classified using the *International Classification of Diseases, Tenth Revision*. Drug overdoses are identified using underlying cause-of-death codes X40–X44 (unintentional), X60–X64 (suicide), X85 (homicide), and Y10–Y14 (undetermined). ^§§^ Drug overdose deaths, as defined, that involve synthetic opioids other than methadone (T40.4). ^¶¶^ Drug overdose deaths, as defined, that involve psychostimulants with abuse potential (T43.6). *** Because deaths might involve more than one drug, some deaths are included in more than one category. In 2019, 6.3% of drug overdose deaths did not include information on the specific type of drug(s) involved.

From 2018 to 2019, the age-adjusted synthetic opioid-involved death rate increased 15.2%, from 9.9 to 11.4. In 2019, the Northeast had the highest percentage and rate of deaths involving synthetic opioids, but the smallest relative (5.2%) and absolute (1.0) rate increases from the previous year (19.1 in 2018 to 20.1 in 2019). In contrast, the West experienced the largest relative (67.9%) and absolute (1.9) rate increases from 2.8 in 2018 to 4.7 in 2019. From 2018 to 2019, a total of 20 states experienced relative increases in their synthetic opioid-involved death rate, with the highest rate in 2019 in Delaware (38.4). The largest relative rate increase occurred in Colorado (95.5%), and the largest absolute rate increase occurred in the District of Columbia (7.6). No state experienced a significant decrease.

The percentage of deaths involving psychostimulants was highest in the West (43.5%) and lowest in the Northeast (7.9%) in 2019. The same geographic pattern was observed with psychostimulant-involved deaths that did not coinvolve synthetic opioids. In all northeastern states, fewer than 20% of drug overdose deaths involved psychostimulants. In 12 states, mostly in the West and Midwest, ≥40% of overdose deaths involved psychostimulants. Among these, the percentage was highest in Hawaii (70.2%) and Oklahoma (50.7%). The percentage was lowest in Maryland (3.3%).

From 2018 to 2019, the age-adjusted rate of psychostimulant-involved deaths increased 28.2%, from 3.9 to 5.0. The Northeast experienced the largest relative (43.8%), but smallest absolute (0.7), rate increase. The Midwest (36.1%) and South (32.4%) experienced similar relative but slightly larger absolute (1.3 and 1.2, respectively) rate increases. Although the percentage of 2019 drug overdose deaths involving psychostimulants was highest in the West, the relative rate increase (17.5%) was lowest there. Twenty-four states experienced an increase in the rate of psychostimulant-involved deaths. Kansas experienced the largest relative increase (107.1%) and third largest absolute rate increase (3.0). West Virginia had the highest 2019 rate (24.4) and the largest absolute rate increase (5.1); New York had the lowest 2019 rate (1.3). No state had a significant decrease (Supplementary Table, https://stacks.cdc.gov/view/cdc/101757).

## Discussion

In 2019, a total of 70,630 drug overdose deaths occurred in the United States; approximately one half involved synthetic opioids. From 2013 to 2019, the age-adjusted synthetic opioid death rate increased sharply by 1,040%, from 1.0 to 11.4. Death rates involving prescription opioids and heroin increased in the presence of synthetic opioids (from 0.3 to 1.8 and from 0.1 to 2.7, respectively), but not in their absence. Death rates involving psychostimulants increased 317% overall, regardless of synthetic opioid coinvolvement. Synthetic opioid- and psychostimulant-involved deaths shifted geographically from 2018 to 2019. From 2015 to 2016, states in the East had the largest increases in deaths involving synthetic opioids, and from 2016 to 2017, the Midwest had the largest increases in deaths involving psychostimulants (*2*,*4*). In contrast, from 2018 to 2019, the largest relative increase in death rates involving synthetic opioids occurred in the West (67.9%); the largest relative increase in death rate involving psychostimulants occurred in the Northeast (43.8%).

Sharp increases in synthetic opioid- and psychostimulant-involved overdose deaths in 2019 are consistent with recent trends indicating a worsening and expanding drug overdose epidemic (*1*,*2*,*4*–*6*). Synthetic opioids, particularly illicitly manufactured fentanyl and fentanyl analogs, are highly potent, increasingly available across the United States, and found in the supplies of other drugs (*7*,*8*). Co-use of synthetic opioids with other drugs can be deliberate or inadvertent (i.e., products might be adulterated with illicitly manufactured fentanyl or fentanyl analogs unbeknownst to the user). Similarly, psychostimulant-involved deaths are likely rising because of increases in potency, availability, and reduced cost of methamphetamine in recent years (*9*). The increase in synthetic-opioid involved deaths in the West and in psychostimulant-involved deaths in the Northeast signal broadened geographic use of these substances, consistent with increases in the number of drug submissions to forensic laboratories in those regions during 2018–2019 (*8*).

The findings in this report are subject to at least two limitations. First, forensic toxicology testing protocols varied by time and jurisdiction, particularly for synthetic opioids. Therefore, some of the increases in overdose deaths reported by drug categories could be attributed to the increases in testing as well as the use of more comprehensive tests. Second, geographic analyses excluded states with inadequate drug specificity or too few deaths to calculate stable rates.

The worsening and expanding drug overdose epidemic in the United States now involves potent synthetic drugs, often in combination with other substances, and requires urgent action. As involved substances and geographic trends in drug overdose deaths change, timely surveillance and evidence-based prevention and response strategies remain essential. CDC’s Overdose Data to Action^¶¶^ cooperative agreement funds health departments in 47 states, the District of Columbia, two territories, and 16 cities and counties to obtain high-quality, comprehensive, and timely data on fatal and nonfatal drug overdoses to inform prevention and response efforts. To help curb this epidemic, Overdose Data to Action strategies focus on enhancing linkage to and retention in substance use disorder treatment, improving prescription drug monitoring programs, implementing postoverdose protocols in emergency departments, including naloxone provision to patients who use opioids or other illicit drugs, and strengthening public health and public safety partnerships, enabling data sharing to help inform comprehensive interventions.*** Other approaches^†††^ should include expanded naloxone distribution and education that potent opioids might require multiple doses of naloxone, improved access to substance use disorder treatment (including medications for opioid use disorder or programs addressing polysubstance use), expanded harm reduction services, and continued partnerships with public safety to monitor trends in the illicit drug supply, including educating the public that drug products might be adulterated with fentanyl or fentanyl analogs unbeknownst to users. A comprehensive and coordinated approach from clinicians, public health, public safety, community organizations, and the public must incorporate innovative and established prevention and response strategies, including those focused on polysubstance use.

**Summary**

**What is already known about this topic?**

Deaths involving synthetic opioids other than methadone, cocaine, and psychostimulants have increased in recent years.

**What is added by this report?**

From 2013 to 2019, the age-adjusted rate of deaths involving synthetic opioids other than methadone increased 1,040%, and for psychostimulants increased 317%. During 2018–2019, the largest relative increase in synthetic opioid-involved death rates occurred in the West (67.9%), and the largest relative increase in psychostimulant-involved death rates occurred in the Northeast (43.8%).

**What are the implications for public health practice?**

Evidence-based prevention and response strategies, including substance use disorder treatment and overdose prevention and response efforts focused on polysubstance use, must be adapted to address the changing drug overdose epidemic.

## Footnotes

† A death involving prescription opioids and heroin would be included in the prescription opioid and heroin death counts and rates.

§ Age-adjusted death rates were calculated by applying age-specific death rates to the 2000 U.S. Census standard population age distribution and are reported per 100,000 population. https://www.cdc.gov/nchs/data/nvsr/nvsr61/nvsr61_04.pdf

¶ *Northeast*: Connecticut, Maine, Massachusetts, New Hampshire, New Jersey, New York, Pennsylvania, Rhode Island, and Vermont; *Midwest*: Illinois, Indiana, Iowa, Kansas, Michigan, Minnesota, Missouri, Nebraska, North Dakota, Ohio, South Dakota, and Wisconsin; *South*: Alabama, Arkansas, Delaware, District of Columbia, Florida, Georgia, Kentucky, Louisiana, Maryland, Mississippi, North Carolina, Oklahoma, South Carolina, Tennessee, Texas, Virginia, and West Virginia; *West*: Alaska, Arizona, California, Colorado, Hawaii, Idaho, Montana, Nevada, New Mexico, Oregon, Utah, Washington, and Wyoming.

** State-level analyses of the percentage of drug overdose deaths involving synthetic opioids excluded one state and involving psychostimulants excluded two states that did not meet the following criteria: >80% of drug overdose death certificates named at least one specific drug in 2019 and ≥10 deaths occurred in 2019 in the specific drug category.

†† State-level analyses comparing death rates from 2018 to 2019 excluded nine states that did not meet the following criteria: >80% of drug overdose certificates named at least one specific drug in 2018 and 2019 and ≥20 deaths occurred during 2018 and 2019 in the drug category examined.

§§ Z-tests were used if the number of deaths was ≥100, and p<0.05 was considered to be statistically significant. Nonoverlapping confidence intervals based on the gamma method were used if the number of deaths was <100 in 2018 or 2019. The method of comparing confidence intervals is a conservative method for statistical significance; caution should be exercised when interpreting a nonsignificant difference when the lower and upper limits being compared overlap only slightly. https://www.cdc.gov/nchs/data/NVSR/NVSR61/NVSR61_04.pdf

¶¶
https://www.cdc.gov/drugoverdose/od2a/index.html

***
https://www.cdc.gov/drugoverdose/pubs/featured-topics/evidence-based-strategies.html

†††
https://emergency.cdc.gov/han/2020/han00438.asp
